# Evaluating the Role of Indocyanine Green Fluorescence Imaging in Enhancing Safety and Efficacy During Laparoscopic Cholecystectomy: A Systematic Review

**DOI:** 10.7759/cureus.73388

**Published:** 2024-11-10

**Authors:** Mina Manasseh, Heather Davis, Kirk Bowling

**Affiliations:** 1 General Surgery, Torbay and South Devon NHS Foundation Trust, Torquay, GBR; 2 Upper Gastrointestinal Surgery, Torbay and South Devon NHS Foundation Trust, Torquay, GBR

**Keywords:** bile duct injury, indocyanine green (icg), laparoscopic cholecystectomy (lc), operative time, visualization of biliary structures

## Abstract

Laparoscopic cholecystectomy (LC) is the standard treatment for gallbladder disease, offering less invasive treatment and quicker recovery. However, bile duct injury (BDI) remains a critical complication. Indocyanine green (ICG) fluorescence imaging has emerged as a valuable technique to improve biliary structure visualization and potentially reduce BDI during LC. This systematic review assesses the efficacy of ICG in reducing BDI over the past decade. A comprehensive search of studies comparing ICG fluorescence and conventional white light (WL) in LC identified 14 studies. Key outcomes such as operative time, incidence of BDI, and visualization of biliary anatomy were analyzed. The results indicate that ICG significantly reduced operative times in complex cases, with an average reduction of approximately 20 minutes compared to WL (p<0.0001). In routine cases, no significant difference in operative time was observed between the two methods. ICG consistently enhanced visualization of key biliary structures, such as the cystic duct and common bile duct, with the greatest benefits seen in anatomically challenging cases. Although the overall incidence of BDI was low, use of ICG showed a trend toward lower BDI rates compared to WL, though the difference was not statistically significant. In conclusion, the use of ICG fluorescence in LC offers notable advantages, particularly in improving visualization of biliary anatomy and reducing operative time in complex cases. While the overall reduction in BDI rates may appear marginal, the clinical importance of avoiding even a single BDI should not be understated, given the serious complications associated with BDI. Our review suggests that the benefits of ICG are most pronounced in more complex cases where biliary anatomy is challenging to identify.

## Introduction and background

Laparoscopic cholecystectomy (LC) is the gold standard treatment for gallbladder disease due to its minimally invasive nature and quicker recovery times compared to open surgery [1]. However, LC has a higher risk of bile duct injury (BDI), a complication that can result in severe morbidity, prolonged hospitalization, and even mortality. The occurrence of BDI can also result in additional complex surgical interventions and a decrease in the patient's quality of life [2].

BDI occurs often due to mistaken identification of biliary structures. Strasberg’s critical view of safety (CVS) technique has been widely adopted as a method to reduce BDI by ensuring proper identification of key biliary structures, specifically the cystic duct (CD) and cystic artery, before any cutting or clipping is performed [3]. Misidentification of the common bile duct (CBD) or other vital structures such as the CD is a common cause of BDI. By providing a clear, standardized approach to visualize biliary anatomy, CVS has become essential in preventing such injuries.

Traditional intraoperative cholangiography (IOC) is a widely used imaging technique during LC to establish CVS and visualize the biliary anatomy, hence reducing the risk BDI [4]. It involves the injection of a contrast dye into the CD, followed by X-ray imaging to map out the bile ducts. By providing a detailed view of the biliary tree, IOC helps surgeons confirm the correct anatomy before proceeding with dissection, thus reducing the risk of misidentifying structures such and the CBD as the CD, which is a common cause of BDI.

However, while IOC can be effective in demonstrating anatomy, it has certain limitations that make it less than ideal in some cases. Firstly, the procedure is often time-consuming because it requires the setup of fluoroscopy equipment, dye injection, and X-ray interpretation during surgery, which can extend operative time. Additionally, IOC requires a certain level of technical skill, and improper execution can lead to inadequate imaging, making it difficult to interpret the anatomy clearly. There is also a small risk associated with the use ionizing radiation and contrast agents, particularly in patients with allergies or renal impairment.

In recent years, indocyanine green (ICG) fluorescence imaging has emerged as an alternative tool to enhance the visualization of biliary structures during LC [5]. ICG is a fluorescent dye that, when injected intravenously, is preferentially taken up by the liver and excreted into the bile ducts. When exposed to near-infrared light, ICG causes the biliary structures, such as the CD, CBD, and CA, to fluoresce, making them more distinguishable from surrounding tissues thereby facilitating real-time visualization of biliary structures during the dissection of Calot's triangle [5]. The timing of ICG injection is critical to ensure that the biliary anatomy lights up distinctly without interference from non-biliary structures [6].

However, the routine use of ICG fluorescence imaging in LC has not yet been standardized, and there is ongoing debate about whether its widespread adoption would significantly reduce the incidence of BDI and improve patient outcomes. This systematic review aims to provide a comprehensive evaluation of the efficacy and safety of ICG fluorescence imaging in LC, specifically comparing its impact on the incidence of BDI to that of conventional white light (WL) imaging.

## Review

Methods

This systematic review was conducted on studies published in English between January 2014 and June 2024, identified using PubMed and EMBASE. The search was carried out using specific MeSH terms: “Indocyanine green”, “laparoscopic cholecystectomy”, and “bile duct injury”. The review focused on studies that compared ICG fluorescence imaging with traditional WL in LC, evaluating outcomes mainly operative times, the incidence of BDI, and the visualization of biliary anatomy.

Data extraction and analysis

Two independent reviewers screened the titles, abstracts, and full texts of the identified studies based on the inclusion and exclusion criteria (Table 1). Any discrepancies were resolved through discussion or consultation with a third reviewer. The extracted data included study design, level of evidence, sample size, ICG dosage, operative time, and biliary anatomy visualization. 

**Table 1:** Inclusion and exclusion criteria BDI, bile duct injury; CA, cystic artery; CBD, common bile duct; CD, cystic duct; ICG, indocyanine green; LC, laparoscopic cholecystectomy; WL, white light

Inclusion criteria	Exclusion criteria
Studies evaluating ICG use in LC	Studies focusing exclusively on robotic cholecystectomy
Studies comparing outcomes of ICG fluorescence imaging with WL	Studies without full-text availability in English
Studies reporting on at least one of the following outcomes: incidence of BDI, visualization of biliary anatomy (CD, CBD, CA), operative time	Pediatric studies, non-human studies, and studies using non-IV administration of ICG
Studies with a sample size of 50 patients or more	Editorials, reviews, case reports, letters, commentaries, and book chapters were also excluded

**Figure 1:**
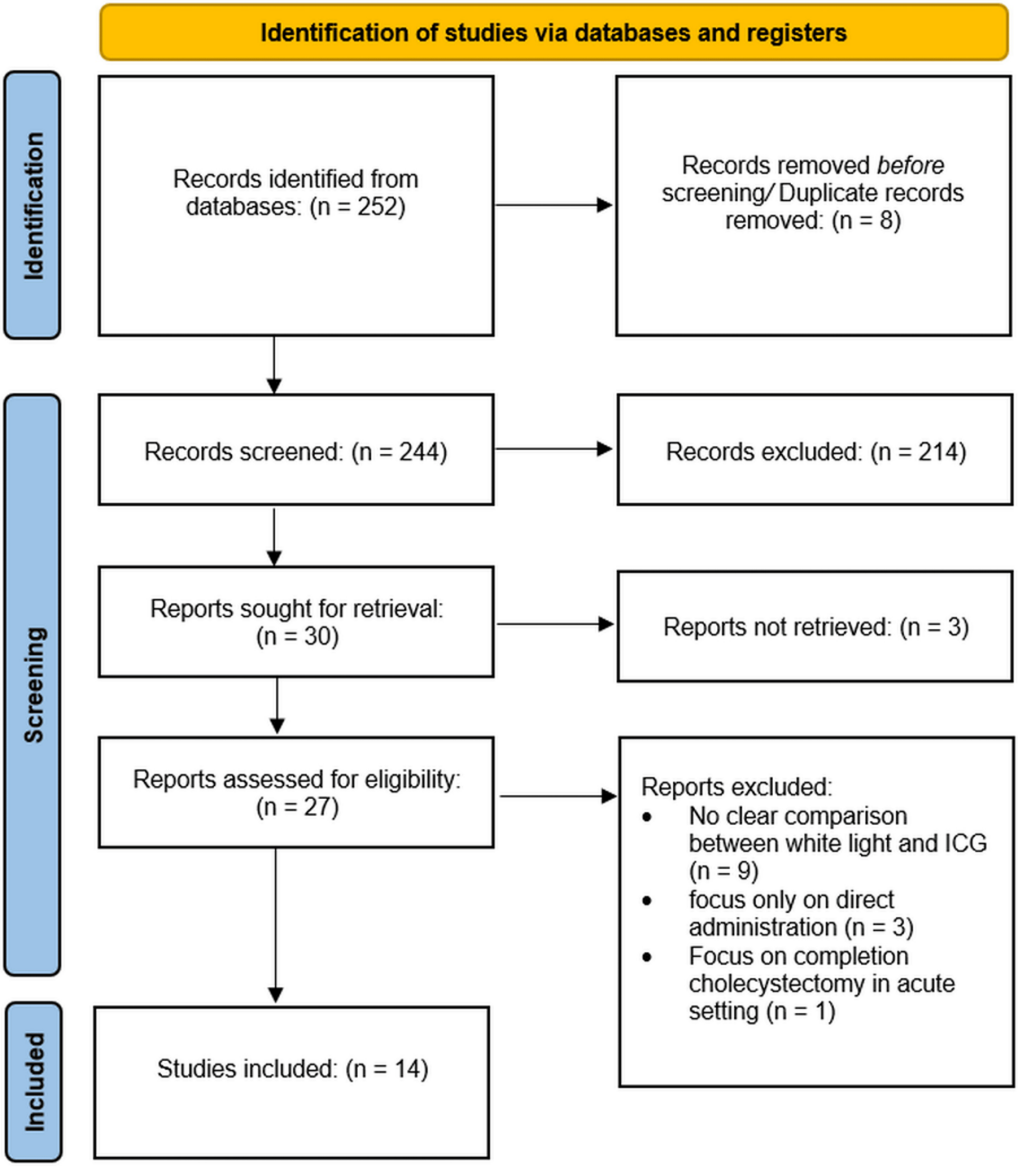
PRISMA flowchart of the studies included PRISMA, Preferred Reporting Items for Systematic reviews and Meta-Analyses

An initial search yielded 249 articles, and after the removal of duplicates and screening for eligibility, 14 studies were included in this review as illustrated in the PRISMA (Preferred Reporting Items for Systematic reviews and Meta-Analyses) flowchart (Figure 1). These studies collectively examined more than 13,000 patients undergoing LC. The included studies consisted of a mix of randomized controlled trials (RCTs), retrospective cohort studies, and meta-analyses (Table 2), providing a comprehensive dataset for evaluating the efficacy of ICG in reducing BDI and improving visualization during surgery.

**Table 2:** Characteristics of included studies comparing ICG fluorescence and WL imaging in LC with level of evidence ICG, indocyanine green; RCT, randomized controlled trial; WL, white light

Author(s)	Year	Country	Study design	Level of evidence
Symeonidis et al. [7]	2024	Greece	RCT	1
Ma et al. [8]	2023	China	Retrospective cohort	3
Xu et al. [9]	2023	China	Retrospective cohort	3
Stolz et al. [10]	2023	USA	RCT	1
Lie et al. [11]	2023	Indonesia	Meta-analysis (22 Studies)	1
Losurdo et al. [12]	2022	Italy	Retrospective cohort	3
Iacuzzo et al. [13]	2022	Italy	Retrospective cohort	3
Jin et al. 2022 [14]	2022	China	Retrospective cohort	3
Lim et al. [15]	2021	Singapore	Meta-analysis (7 studies)	2
Dip et al. [16]	2021	Argentina, Canada, USA	Meta-analysis (16 studies)	1
Broderick et al. [17]	2021	USA	Retrospective cohort	3
Keeratibharat [18]	2021	Thailand	Retrospective cohort	3
Ambe et al. [19]	2019	Germany	Retrospective cohort	3
Dip et al. [20]	2019	USA, Germany, Italy, Argentina, Japan	RCT	1

Results

Operative Time

Analyzing pooled data from all the studies revealed that the weighted average operative time was 75.3 minutes for the ICG group compared to 96.9 minutes for the WL group, indicating a general reduction in operative time with ICG (Table 3). The ICG operative time ranged from 21.3 minutes to 117 minutes, while the WL operative time ranged from 46.1 minutes to 137 minutes. Using data from meta-analyses [11,16] to estimate the pooled standard deviation, the calculated p-value was p<0.0001, indicating a significant reduction in operative time with ICG fluorescence.

**Table 3:** Comparison of operative times using ICG fluorescence vs WL in LC ICG, indocyanine green; LC, laparoscopic cholecystectomy; WL, white light

Author(s) and year	Sample Size	ICG sample size	WL sample size	ICG Dose	ICG operative time	WL operative time
Symeonidis et al. 2024 [7]	160	80	80	0.3 mg/kg	46.5 ± 7.43 min	47.1 ± 7.31 min, p = 0.858
Ma et al. 2023 [8]	131	59	72	2.5-3 mL	117 min (105-145 min)	137 min (115-195 min), p<0.05
Xu et al. 2023 [9]	624	221	403	0.4-1 mL (1-2.5 mg)	52.5 min (40-70 min); in difficult cases: 65 min (45-80 min)	60 min (45-75 min), p = 0.020; in difficult cases: 70 min (60-90 min), p = 0.003
Stolz et al. 2023 [10]	50	50	50	2.5 mg	Not specified	Not specified
Lie et al. 2023 [11]	3457	1886	1571	0.05-2.5 mg/kg	81.24 min	97.3 min, p = 0.007
Losurdo et al. 2022 [12]	120	48	72	0.02-0.25 mg/kg	82.5 ± 34 min	111 ± 42 min, p = 0.002
Lacuzzo et al. 2022 [13]	161	63	98	N/A	61 min (25-160 min)	85 min (20-285 min), p = 0.002
Jin et al. 2022 [14]	238	112	126	0.5 mg/kg (2.5 mg/mL)	21.3 ± 1.2 min	46.1 ± 5.3 min, p = 0.000
Lim et al. 2021 [15]	481	377	377	Varied	78.25 min	81.03 min
Dip et al. 2021 [16]	6673	1603	5070	Varied	Not specified	Not specified
Broderick et al. 2021 [17]	1389	400	989	Not specified	72.53 min	99 min, p<0.0001
Keeratibharat 2021 [18]	55	55	55	2.5 mg	61.7 ± 27.6 min	Same cohort
Ambe et al. 2019 [19]	70	29	41	Not specified	53.0 min (28-140 min)	54 min (30-145 min), p = 0.4
Dip et al. 2019 [20]	639	321	318	0.05 mg/kg	Not specified	Not specified

Overall, in the largest meta-analysis by Lie et al. [11], which included 3,457 patients, it was reported that ICG significantly reduced the operative time compared to WL (81.24 minutes vs 97.3 minutes, p = 0.007), demonstrating a potential advantage of ICG in certain settings. Similarly, Ma et al. [8] reported a significant reduction in operative time by 20 minutes in the ICG group (117 minutes) compared to the WL group (137 minutes, p<0.05), specifically highlighting the benefit of ICG in complex cases with challenging anatomy. Iacuzzo et al. [13] further supported these findings, reporting a significant reduction in operative time when using ICG during complex procedures, with a median time of 61 minutes compared to 85 minutes in the WL group (p = 0.002)

Xu et al. [9], in their study of 624 patients, also showed a statistically significant reduction in operative time for ICG, particularly in difficult cases. In these complex cases, the mean operative time was 65 minutes compared to 70 minutes in the WL group (p = 0.003). While the reduction was less pronounced in routine cases, there was still a significant difference, with a median operative time of 52.5 minutes in the ICG group versus 60 minutes in the WL group (p = 0.020).

Conversely, Symeonidis et al. [7], in an RCT of 160 patients, observed no significant difference in operative times between the ICG group and the WL group (46.5 ± 7.43 minutes vs. 47.1 ± 7.31 minutes, p = 0.858), suggesting that ICG may not provide a substantial time-saving benefit in routine cases.

Visualization of Biliary Anatomy

Across the studies included, ICG fluorescence consistently demonstrated superior visualization of key biliary structures compared to WL (Table 4). Accordingly, the study data suggests that the benefits of ICG would be more evident in difficult cases. When pooling the data, ICG significantly improved visualization of the CD and CBD in the majority of studies.

**Table 4:** Comparison of visualization of biliary structures and incidence of BDI using ICG fluorescence vs WL in LC BDI, bile duct injury; CBD, common bile duct; CD, cystic duct; CHD, common hepatic duct; ICG, indocyanine green; LC, laparoscopic cholecystectomy; WL, white light; -, not specified

Author(s) and year	Visualization of CD	Visualization of CBD	Visualization of CHD	Visualization of the CD-CBD junction	Incidence of BDI using ICG	Incidence of BDI using WL
Symeonidis et al., 2024 [7]	No significant difference (p = 0.225)	No significant difference (p = 0.276)	No significant difference (p = 0.940)	No significant difference (p = 0.827)	0	0
Ma et al., 2023 [8]	Before dissecting Calot’s: no significant difference (p = 0.075). After dissecting Calot’s: ICG significantly improved visualization (p = 0.02)	Before dissecting Calot’s: no significant difference (p = 0.075). After dissecting Calot’s: ICG significantly improved visualization (p = 0.02)	-	-	0	0
Xu et al., 2023 [9]	-	-	-	-	0	0
Stolz et al., 2023 [10]	No significant difference	No significant difference	No significant difference	No significant difference	-	-
Lie et al., 2023 [11]	Improved RR 1.24, 95% CI 1.07–1.43, p = 0.003	Improved: RR 1.31, 95% CI 1.07–1.60, p = 0.009	-	-	No significant difference: (RR 0.34, 95% CI 0.07–1.58, p = 0.17)	No significant difference: (RR 0.34, 95% CI 0.07–1.58, p = 0.17)
Losurdo et al., 2022 [12]	-	-	-	-	0	1.4%, p = 0.728
Lacuzzo et al., 2022 [13]	-	-	-	-	0	0
Jin et al., 2022 [14]	-	-	-	-	0	1.83%, p = 0.389
Lim et al., 2021 [15]	No significant difference: RR = 0.90, p = 0.12, 95% CI 0.79– 1.03, I² = 74%	No significant difference: RR = 0.82, p = 0.09, 95% CI 0.65– 1.03, I² = 87%	ICG significantly improved visualization: RR = 0.58, p = 0.03, 95% CI 0.35–0.93, I² = 91%	No significant difference: RR = 0.68, p = 0.06, 95% CI 0.45– 1.02, I² = 94%	0	2 (0.55%)
Dip et al., 2021 [16]	-	-	-	-	1 (0.06%)	12 (0.25%)
Broderick et al., 2021 [17]	-	-	-	-	0	1 (0.1%), p = 1
Keeratibharat, 2021 [18]	ICG significantly improved visualization, p = 0.001	ICG significantly improved visualization, p = 0.002	ICG significantly improved visualization, p = 0.000	-	0	0
Ambe et al., 2019 [19]	-	-	-	-	0	0
Dip et al., 2019 [20]	Before dissecting Calot’s: ICG significantly improved visualization (p ≤ 0.001). After dissecting Calot’s: no significant difference (p = 0.83)	Before and after dissecting Calot’s: ICG significantly improved visualization (p < 0.001)	Before and after dissecting Calot’s: ICG significantly improved visualization (p < 0.001)	Before and after dissecting Calot’s: ICG significantly improved visualization (p < 0.001)	0	2 (0.62%)

For the CD, Lie et al. [11] reported a relative risk improvement of 1.24 (95% CI 1.07-1.43, p = 0.003), indicating that ICG was notably more effective than WL. Similarly, Keeratibharat [18] found that ICG significantly improved CD visualization (p = 0.001). Ma et al. [8] also observed superior visualization of the CD after dissecting Calot’s triangle (p = 0.02). In contrast, Symeonidis et al. [7] found no significant difference between the two modalities in routine cases (p = 0.225).

For the CBD, multiple studies supported the enhanced visualization offered by ICG. Lie et al. [11] showed an improved relative risk of 1.31 (p = 0.009) for CBD visualization, while Keeratibharat [18] also found a statistically significant improvement (p = 0.002). However, Lim et al. [15] and Symeonidis et al. [7] found no significant differences in CBD visualization.

The visualization of other critical structures, such as the common hepatic duct (CHD) and the CD-CBD junction, also showed some variability. Lim et al. [15] demonstrated significantly improved visualization of the CHD (RR = 0.58, p = 0.03), while Symeonidis et al. [7] found no significant difference for these structures.

Risk of Bile Duct Injury

Across the studies, the overall incidence of BDI was low, but there were differences in outcomes between the ICG and WL groups (Table 4). Despite these differences, statistical significance was not achieved due to the relatively rare occurrence of BDI. The weighted average BDI incidence for the ICG group was approximately 0.018%, while the WL group had a higher weighted average incidence of approximately 0.225%.

In the meta-analysis by Lie et al. [11], which pooled data from 22 studies, the incidence of BDI was lower in the ICG group compared to WL, though this finding was not statistically significant (p = 0.17). Dip et al. [20] provided further evidence of a reduction in BDI with ICG. In their cohort, BDI occurred in only two (0.06%) patients in the WL group, while no injury in the ICG group. Broderick et al. [17] reported no significant difference in BDI between the two groups. In this study, BDI occurred in one (0.1%) patient in the WL groups (p = 1.0).

Studies focusing on complex cases, where ICG theoretically provides the greatest benefit, showed mixed results. Losurdo et al. [12] and Jin et al. [14] both reported 0% BDI in the ICG groups, compared to 1.4% and 1.83% in the WL groups, respectively. However, neither study reached statistical significance, and the small sample sizes and low event rates further limited the findings.

Discussion 

ICG is a safe adjunct to LC, with a minimal side effect profile and without the toxicities or drawbacks of conventional imaging methods [21]. ICG’s real-time visualization capabilities offer benefits across various specialties. In colorectal surgery, ICG fluorescence angiography has been shown to reduce anastomotic leakage by enabling precise visualization of bowel perfusion [22,23]. Similarly, in plastic and reconstructive surgery, ICG angiography provides accurate insights into flap perfusion in complex procedures such as free flap breast reconstruction, which has been shown to reduce fat necrosis [24].

Our study demonstrated that ICG has benefits in complex LC in reducing operative time and improving visualization of critical structures. This has potential benefits in reducing complications, namely BDI. When considering alternative imaging modalities, ICG fluorescence cholangiography has been shown to be non-inferior to X-ray cholangiography in visualizing critical biliary junctions, particularly in challenging cases [25]. Similarly, intraoperative ultrasound (IOUS) cholangiography is a highly effective adjunct in difficult biliary surgeries [26], with high sensitivity (90%-96%) and specificity (up to 100%) in identifying biliary structures, thereby reducing the risk of BDI [27]. The primary advantages of IOUS include the absence of ionizing radiation, quicker execution, lower failure rates, and the ability to be repeated as needed during the procedure [28]. However, IOUS requires probe manipulation and depends significantly on the operator's skill, which may introduce variability and disrupt the laparoscopic workflow. In contrast, ICG fluorescence integrates directly with laparoscopic systems, providing continuous real-time visualization without additional handling or workflow disruption, making it particularly valuable in cases where a stable view of biliary anatomy is essential. Although ICG’s rapid dye clearance can limit its reusability within a single procedure, it remains an effective tool that offers practical advantages, especially in anatomically challenging cases. Notably, no large-scale studies to date have directly compared ICG with IOUS, despite calls from experts [29].

The impact of ICG fluorescence on operative time varied across the studies reviewed. In straightforward cases with clear anatomical landmarks, ICG’s effect on reducing operative time was minimal. This suggests that ICG may not offer substantial time-saving advantages when anatomy is uncomplicated and easily identifiable. However, in difficult scenarios, such as cases involving inflammation, obesity, or anatomical variance, ICG fluorescence demonstrated a greater impact on reducing operative time by clarifying anatomy and allowing more efficient dissection.

Notably, the delayed elective surgery in the aftermath of COVID-19 has resulted in patients presenting with more advanced and complicated gallbladder disease than previously. Prolonged waiting times have exacerbated conditions such as chronic cholecystitis, as well as increasing inflammation and scarring, which further obscures the biliary anatomy during surgery [30,31]. In these challenging cases, ICG fluorescence can be particularly valuable by offering enhanced visualization and reducing operative time during more difficult dissections.

Although ICG shows the greatest significance in complex cases, routine use may be beneficial to facilitate familiarity with the technology and improve the surgical team's confidence and accuracy in interpreting ICG imaging. Using ICG in simpler cases could allow more rapid progression along the learning curve, making it easier to use in complex cases without added cognitive burden.

Widespread implementation of ICG may not be universally cost-effective; however, given the increased incidence of complex cases and the demonstrated time savings, ICG presents a potential financial asset. The cost of running an operating theatre is approximately £1,200 an hour [32], and LC is one of the most common general surgical operations, with more than 66,000 performed per year [33]. Moreover, Reeves et al. [34] identified fluorescent cholangiography to reduce lifetime costs by approximately $1,235 per patient compared to standard LC, primarily through shorter operative times and a lower conversion rate to open surgery (p < 0.0001). While these findings are promising, further large-scale studies are needed to fully establish the cost-effectiveness of ICG across various clinical settings and patient populations.

This study clearly demonstrates that ICG fluorescence has clear advantages over traditional WL imaging in visualizing critical biliary structures, such as the CD, CBD, and cystic artery. This enhanced clarity is particularly valuable in anatomically complex cases where conventional methods such as the CVS may be insufficient. By providing real-time delineation of these structures, ICG fluorescence can help mitigate the risk of misidentification that may lead to complications.

Overall, while the current data suggest that ICG fluorescence may reduce the risk of BDI, the studies were not sufficiently powered to show a statistically significant reduction in BDI rates due to the low incidence of BDI. Future studies with larger cohorts may provide more definitive evidence regarding the protective effects of ICG in preventing BDI during LC.

Caution is needed, as the assumption that improved visualization directly causes reduced rates of BDI requires further investigation. Enhanced imaging aids structural identification, but it does not eliminate the inherent risks of surgery. An over-reliance on ICG could lead to excessive confidence in anatomical identification, increasing the risk of errors.

We believe that ICG should complement, not replace, meticulous surgical technique and sound clinical judgment. BDI is a life-changing event. Indeed, long-term studies indicate that BDI-related mortality ranges from 1.8% to 4.6% [2]. Despite reported successful clinical outcomes of endoscopic, radiologic, and surgical treatments of BDI, the long-term impact on quality of life may remain impaired, even after clinically successful treatment [2]. Surgeons must remain cautious and not depend too heavily on ICG, as no technology can substitute experience and careful decision-making during complex dissections.

## Conclusions

In conclusion, the use of ICG fluorescence in LC offers notable advantages, particularly in improving visualization of biliary anatomy and reducing operative time in complex cases. While the overall reduction in BDI rates does not reach statistical significance, the associated morbidity and mortality convey very real significance to both patients and surgeons. This review suggests that the benefits of ICG are most pronounced in cases where biliary anatomy is challenging. For routine cases, the advantages of ICG over WL may be less pronounced, and the decision to use ICG could be based on surgeon experience and patient-specific considerations.

Future research should prioritize large-scale RCTs as well as the development of standardized ICG dosing protocols to better define ICG’s role in reducing BDI across diverse patient populations and varying levels of surgical complexity. Additionally, exploring the cost-effectiveness of integrating ICG into standard practice will provide valuable insights to support its broader adoption.
